# Microglial Cell Morphology and Phagocytic Activity Are Critically Regulated by the Neurosteroid Allopregnanolone: A Possible Role in Neuroprotection

**DOI:** 10.3390/cells10030698

**Published:** 2021-03-21

**Authors:** Valérie Jolivel, Susana Brun, Fabien Binamé, Jérémie Benyounes, Omar Taleb, Dominique Bagnard, Jérôme De Sèze, Christine Patte-Mensah, Ayikoe-Guy Mensah-Nyagan

**Affiliations:** Biopathologie de la Myéline, Neuroprotection et Stratégies Thérapeutiques, INSERM U1119, Centre de Recherche en Biomédecine de Strasbourg (CRBS), Fédération de Médecine Translationnelle de Strasbourg (FMTS), Université de Strasbourg, 1 rue Eugène Boeckel, 67000 Strasbourg, France; vjolivel@unistra.fr (V.J.); susana.brun@unistra.fr (S.B.); biname@unistra.fr (F.B.); jbenyounes@unistra.fr (J.B.); omar@unistra.fr (O.T.); bagnard@unistra.fr (D.B.); jerome.de.seze@chru-strasbourg.fr (J.D.S.); cmensah@unistra.fr (C.P.-M.)

**Keywords:** microglia, neurosteroid, allopregnanolone, GABA-A receptor, oligodendrocyte, multiple sclerosis

## Abstract

Microglial cells are key players in neural pathogenesis and microglial function regulation appears to be pivotal in controlling neuroinflammatory/neurological diseases. Here, we investigated the effects and mechanism of action of neurosteroid allopregnanolone (ALLO) on murine microglial BV-2 cells and primary microglia in order to determine ALLO-induced immunomodulatory potential and to provide new insights for the development of both natural and safe neuroprotective strategies targeting microglia. Indeed, ALLO-treatment is increasingly suggested as beneficial in various models of neurological disorders but the underlying mechanisms have not been elucidated. Therefore, the microglial cells were cultured with various serum concentrations to mimic the blood-brain-barrier rupture and to induce their activation. Proliferation, viability, RT-qPCR, phagocytosis, and morphology analyzes, as well as migration with time-lapse imaging and quantitative morphodynamic methods, were combined to investigate ALLO actions on microglia. BV-2 cells express subunits of GABA-A receptor that mediates ALLO activity. ALLO (10µM) induced microglial cell process extension and decreased migratory capacity. Interestingly, ALLO modulated the phagocytic activity of BV-2 cells and primary microglia. Our results, which show a direct effect of ALLO on microglial morphology and phagocytic function, suggest that the natural neurosteroid-based approach may contribute to developing effective strategies against neurological disorders that are evoked by microglia-related abnormalities.

## 1. Introduction

Microglia represent the resident macrophages of the central nervous system (CNS) [1]. They are derived from erythromyeloid progenitors that are found in the yolk bag and they are maintained throughout life without the contribution of adult hematopoiesis [2]. Under physiological conditions, microglia are distributed throughout the whole CNS, where they perform tasks that are essential to development and homeostasis. Their thin and ramified processes are continuously scanning the environment [3,4]. Upon detection of an activating stimulus in the surrounding environment, microglia rapidly change their morphology, may adopt an inflammatory or an anti-inflammatory polarization, and actively perform debris phagocytosis [2].

Multiple sclerosis (MS) is a major neurological disorder involving microglia. MS is an autoimmune disease that leads to demyelination of the CNS. MS represents the most common non-traumatic neurological disorder in young patients. It is characterized by the presence of focal demyelinating lesions that exhibit variable degrees of inflammation, reactive gliosis, and neurodegeneration [5]. A hallmark of this pathology is the presence of myelin-laden phagocytes [6]. In normal-appearing white matter of MS patients, clusters of reactive microglia are found in the absence of leukocyte infiltration and demyelination [7]. These nodules, expressing high levels of pro-inflammatory genes and considered as ‘pre-active lesions’, may eventually develop into active demyelinating MS lesions [8]. A similar observation has been done in experimental autoimmune encephalomyelitis (EAE), a commonly used animal model of MS. At early stages of the disease, i.e., before paralysis onset, microglia form perivascular nodules triggered by fibrinogen leakage [9]. Interestingly, focal rupture of the blood-brain barrier (BBB) also occurs in patients with progressive MS [10]. The exposure of microglia to serum leads to functional and transcriptomic modifications. In particular, they adopt a round amoeboid shape, a representative morphology of activated microglia, and they increase their phagocytic activity [11]. These data suggest that early BBB permeabilization may trigger microglia activation upstream formation of new lesions in MS.

Recently, a large body of proof has highlighted the important cross-talk occurring between the nervous and immune systems. Intriguingly, neural and immune cells share components of the neurotransmitters pathways. Accordingly, immune cells, such as lymphocytes, dendritic cells, or macrophages, express the metabolic machinery for γ-aminobutyric acid (GABA), the principal inhibitory neurotransmitter in the adult CNS [12]. This immune GABA signaling system is active and it can modulate various functions, like cytokine secretion, cell proliferation, phagocytic activity, and chemotaxis [12]. Human and mouse microglia, as well as the murine microglial BV-2 cell line, express GABA-A receptors that modulate microglial functions, such as motility and volume sampling [4,13,14]. Moreover, GABA seems to be implicated in autoimmune diseases, like multiple sclerosis [15]. Indeed, the oral administration of agents increasing GABA concentrations delays the onset of paralysis in an EAE model via the inhibition of inflammation [16]. GABA can exert its activity via two classes of receptors: the ionotropic GABA-A receptors and the metabotropic GABA-B receptors, both being expressed by microglia [17,18]. Numerous modulators of the GABA-A receptors have been described, among them the class of neuroactive steroids [19]. Interestingly, a disturbance of neurosteroid synthesis has been reported in the CNS of MS patients, as well as in an EAE model [20,21,22]. In the work of Noorbakhsh et al., measurements by gas chromatography–mass spectrometry revealed reduced levels of dehydroepiandrosterone and ALLO in the brain of MS patients [20]. Interestingly, the administration of ALLO to mice after EAE induction limited the development of the pathology. Besides MS, the neuroprotective activity of ALLO has been shown in many animal models of neurological diseases. such as Niemann-Pick disease [23], Alzheimer’s disease [24], Parkinson’s disease [25], peripheral neuropathies [26,27,28,29], or brain ischemia [30]. In vitro, ALLO exhibited a direct antiapoptotic activity on neurons [31] via a reduction of the release of cytochrome c, the inhibition of Bax translocation, and DNA fragmentation [32]. This neuroprotective effect induced the transcription of GABA-A receptor subunits [33]. The beneficial activity of ALLO is also mediated by a reduced production of reactive oxygen species (ROS) [34]. Besides the direct antiapoptotic effect exerted by ALLO on neurons, the immunomodulatory activities of this neurosteroid, especially on microglia, are poorly known.

The interactions that were observed between neurotransmitters and the immune system represent new prospects for understanding neuroinflammation [35,36]. Targeting microglia would represent a potential new therapeutic strategy for neurological diseases. In this context, we analyzed the effect of ALLO on microglial functions in physiological and pathological conditions. We found that this neurosteroid induces the elongation of the processes that are borne by microglia and modulates their migration, as well as their phagocytic capacity.

## 2. Materials and Methods

### 2.1. Cell Culture and Treatment

The murine microglial BV-2 cell line (that was kindly provided by Prof. H. Luhmann, University of Mainz, Germany) and the murine oligodendroglial 158N cell line (generated in the laboratory) were cultured in DMEM with GlutaMAX (Gibco, Bazel, Switzerland), 4.5 mg/mL glucose, which was supplemented with 50 U/mL penicillin (Gibco), 50 µg/mL streptomycin (Gibco) and 10% (for BV-2) or 5% (for 158N) heat-inactivated Fetal Calf Serum (FCS, Gibco). The cells were subcultured twice a week after their detachment and dissociation with the Cell Dissociation Solution Non-enzymatic 1x (Sigma, Saint-Quentin, France). Primary murine microglia were isolated from C57BL/6J mouse pups. In brief, the brains were harvested from postnatal day 1–4 mice. The cortices were dissected from the forebrain, and the surrounding meninges were removed. Intact cortices were mechanically and enzymatically dissociated while using trypsin. The reaction was stopped by the addition of trypsin inhibitor. Mixed glial cultures were established in DMEM/F12 with GlutaMAX, which was supplemented with 10% FCS and penicillin/streptomycin. After 2 weeks, microglia were isolated and re-plated. Culture purity was assessed by microscopy or flow cytometry after staining with antibodies that were directed against Iba-1, or CD45 and CD11b respectively. Murine peritoneal macrophages were obtained from adult C57BL/6J by peritoneal lavage with 5 mL ice-cold PBS. The cells were plated in RPMI 1640 supplemented with 10% FCS and penicillin/streptomycin. The macrophages were subsequently purified by adherence to tissue culture plastic. All of the cells were grown at 37°C in a humidified incubator with 5% CO2. To treat the cells, stock solutions of ALLO (Sequoia Research Product Ltd., United Kingdom) were prepared with DMSO.

To mimic BBB disruption leading to microglial activation, BV-2 cells and primary murine microglia were cultured in the presence of 10% FCS, whereas more quiescent cells were obtained thanks to incubation with 1% FCS.

### 2.2. Evaluation of Cell Viability

Cell survival is evaluated by either flow cytometry or MTT assay. For the cytometric determination, the BV-2 cells were plated in triplicate into 24-well plates (3 × 10^5^ cells/mL) and then incubated with graded concentrations of ALLO comprised between 0 and 10 µM in presence of 1% or 10% FCS. Both floating and adherent cells were pooled in a hemolysis tube, centrifuged, and then resuspended into 300 µL PBS. Prior to the cytometric analysis, 1 µL of 7-AAD (BioLegend, London, United Kingdom) was added to the cell suspension. The acquisition was realized with the BD Accuri C6 Plus flow cytometer (BD Biosciences, Le Pont de Claix, France). After exclusion of debris and doublets, the percentage of living cells (7-AAD negative cells) was measured. For the MTT assay, BV-2 and 158N cells were seeded at 3 × 10^3^ cells per well into 96-well plates and then incubated with graded concentrations of ALLO comprised between 0 and 50 µM in presence of 1% or 10% FCS. The MTT (Sigma) was added to a final concentration of 0.5 mg/mL to each well and then allowed to incubate in the dark at 37°C for 2 h to obtainpurple colored formazan products. Afterwards, the medium was removed and the formazan crystals were dissolved with 200 µL DMSO. Spectrophotometric measurements were performed at 570 nm to determine the cell viability (Multiskan Go, Thermo Scientific, Villebon sur Yvette, France).

In order to evaluate the deleterious activity of BV-2, the culture medium of 158N seeded in 24-well plates was replaced by supernatants isolated after 24 h of contact with BV-2 cells. One day later, the viability of 158N cells was measured by flow cytometry following a staining with 7-AAD.

### 2.3. Analysis of Apoptotic and Necrotic Cell Death

After incubation with ALLO or vehicle, the BV-2 cells were detached and stained with annexin V coupled to PE and propidium iodide (PI), according to the manufacturer’s protocol (Invitrogen, Saint Aubin, France). The activation of caspase-3 was also investigated with the ‘PE Active Caspase-3 Apoptosis Kit’ (BD Biosciences). The cells were subsequently analyzed by flow cytometry to distinguish the different stages of cell death.

### 2.4. Analysis of Cell Proliferation

BV-2 and 158N cells were labeled with 5 µM of the cell tracer Carboxyfluorescein Diacetate Succinimidyl Ester (CFSE, eBioscience, San Diego, CA, USA) and then cultured in triplicate in 24-well plates. The next day, they were treated with graded concentrations of ALLO or vehicle. After 3–4 days of culture, the cells were detached and washed with PBS. They were subsequently analyzed by flow cytometry. After the exclusion of debris, the gate was applied on the live cell population, the doublets were removed, and the dilution of CFSE was determined.

### 2.5. Phagocytosis Assay

BV-2 cells, primary microglia, Raw264.7, or peritoneal macrophages were seeded in 24-well plates. Twenty-four hours after treatment with graded concentrations of ALLO or vehicle, the cells were incubated with FITC-dextran 70 kDa (Sigma, final concentration: 1 mg/mL) during 2 hat 37°C. The negative control cells were incubated on ice. BV-2 cells were alternatively incubated with 1-µm latex beads coupled to FITC (Sigma). The internalization of FITC-dextran or FITC-latex beads was measured by flow cytometry after detachment and extensive washing of the cells. A double staining with anti-CD11b was performed to assess microglia or macrophage purity.

### 2.6. BV-2 and 158N Cell Line Co-Culture

158N cells were stained with CFSE and plated into 24-well plates (75 × 10^3^ cells/mL). BV-2 cells stably expressing tdTomato have been generated with a lentiviral construct. Twenty-four hours after a treatment with ALLO or vehicle, BV-2 tomato cells grown in the presence of 10% FCS were detached, washed, and suspended in 158N dedicated culture medium. BV-2 cells (50 × 10^3^ cells/well) were added on the top of CFSE-158N cells. After 24-h co-culture, the cells were analyzed by flow cytometry to count the BV-2 cells having phagocytized 158N cells or fixed for microscopic observation.

### 2.7. Morphological Analysis of BV-2 Cells

The BV-2 cells were seeded in 35 mm-Petri dishes. Twenty-four hours after treatment with ALLO or vehicle, the microphotographies were realized using the microscope Eclipse TS100 (Nikon, Champigny sur Marne, France) that was equipped with the DS-FI1 camera (Nikon) with the ×10 or the ×40 objective. The number of primary processes, i.e., those that emerge from the cell body, allows for distinguishing between bipolar and multipolar microglia. Bipolar cells exhibit two primary processes and multipolar cells more than three processes. The total length of each process, from the emergence point to the further extremity, was manually measured using ImageJ.

### 2.8. Morphological Analysis of Primary Microglial Cells

The primary microglial cells were seeded in Lab-TekTM chamber slides (Thermo Scientific Nunc). Twenty-four hours after treatment with ALLO or vehicle, the cells were fixed in 2% paraformaldehyde and subsequently immunostained with a primary antibody against Iba-1 (Wako, Osaka, Japan), followed by a secondary antibody coupled to AlexaFluor 488 (Abcam, Paris, France). The nuclei were counterstained with DAPI. Microphotographies were realized using the microscope IX73 (Olympus, Rungis, France) with the ×20 objective. The total cell length of bipolar Iba-1 positive cells was manually measured using ImageJ.

### 2.9. Time-Lapse Imaging and Quantitative Morphodynamics Analysis of BV-2 Cells 

The BV-2 cells were seeded in 60 mm-Petri dishes at the density of 3 × 10^5^ cells/mL and then treated with 10 µM ALLO or the vehicle (DMSO). The next day, time-lapse imaging was acquired using the microscope Axiovert 200M (Zeiss, Jena, Germany) with the ×10 objective. The system was equipped with the software MetaView. The cells were imaged during 2 h and a brightfield picture was taken every 2 min. The analysis of the movement that was done by the nuclei was performed using the plugin MTrackJ available by ImageJ [35]. The total path traveled by the cells (= path length) during the experiment and the distance between the start and the end point (= D2S) were measured.

### 2.10. Reverse Transcription and Real-Time Quantitative PCR

The NucleoZOL reagent (Macherey-Nagel, Hoerdt, France) was used according to the manufacturer’s instructions for the extraction of total RNA from BV-2 cells that were grown either in the presence of 1% or 10% FCS. Reverse transcription was performed with 1 μg RNA using the iScript™ cDNA synthesis kit (Bio-Rad, Hercules, CA, USA). The quantitative PCR was performed with a CFX96 PCR detection system (Bio-Rad) using Bio-Rad SYBRGreen^®^ dye. In order to calculate starting quantities, standard curve based on successive cDNA dilutions was set up by using the iCycleriQ optical system software (CFX manager Maestro Bio-Rad). Starting quantities of genes of interest were reported to those of the peptidylprolyl isomerase A (Ppia, cyclophilin A), phosphoglycerate kinase 1 (Pgk1) and hydroxymethylbilane synthase (Hmbs) genes used as reference genes. All of the samples were analyzed in triplicate. The specificity of the amplification was controlled by a melting curve ranging from 62°C to 95°C and allowing for the identification of a single peak that corresponds to the amplicon. Table 1 provides the primer sequences used for this study. The primer sequences for Hmbs were designed by Bio-Rad (Assay ID: qMmuCID0022816).

### 2.11. Statistical Analysis

Statistical analysis was performed using GraphPad Prism. The data are presented as mean ± SEM from at least three independent experiments. Statistical analysis of the data was conducted using a non-parametric test (Mann–Whitney or Kruskal–Wallis tests), followed by a Dunn’s multiple comparison test. Statistical analysis of the data from migration studies was realized using a parametric test (unpaired *t*-test).

## 3. Results

### 3.1. Serum Represents an Activation Factor for the BV-2 Cell Line

**Figure 1:**
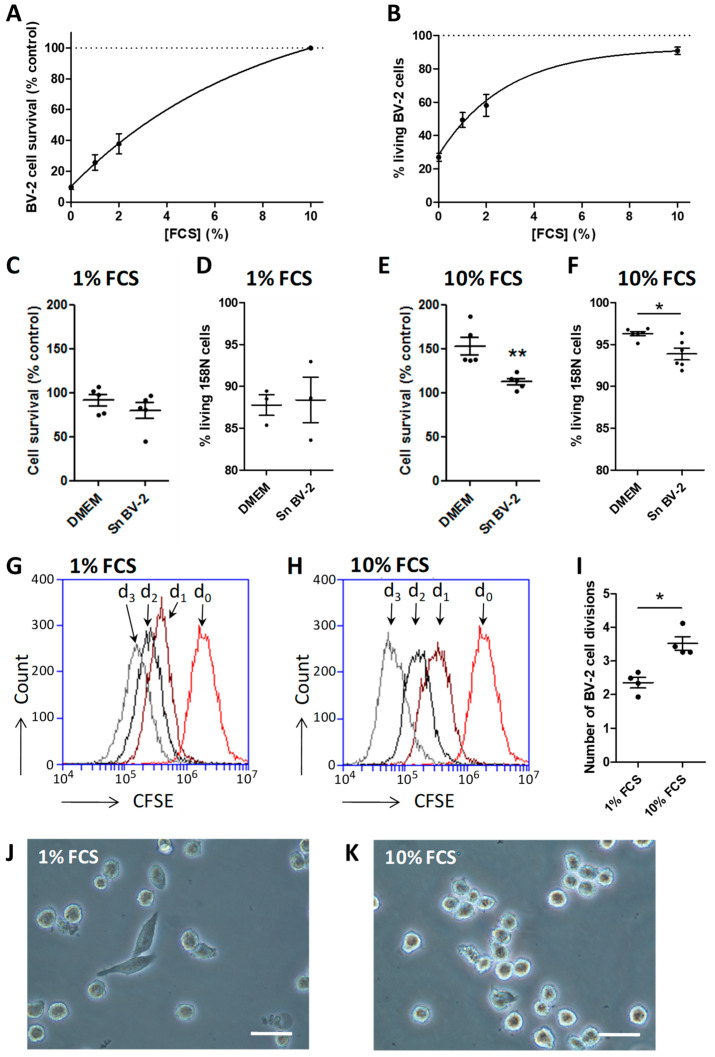
Variations of serum concentrations affect the viability and differentiation of BV-2 cells. (**A**) The viability of BV-2 cells grown in presence of different serum concentrations was assessed by a MTT assay and corroborated (**B**) with a 7-AAD viability staining measured by flow cytometry. (**C**,**D**) The supernatant of BV-2 grown in a low concentration of serum (1% Fetal Calf Serum (FCS)) does not modify the survival of 158N oligodendrocyte cell line, whereas (**E**,**F**) the supernatant of BV-2 grown in a high concentration of serum (10% FCS) reduced significantly the survival of 158N cells. (**G**–**I**) The Carboxyfluorescein Diacetate Succinimidyl Ester (CFSE) dilution was used to determine the proliferation rate of the BV-2 cells. The CFSE dilution was followed day by day by flow cytometry in the culture condition (**G**) 1% FCS or (**H**) 10% FCS. D: day. (**I**) Determination of the number of cell divisions showed that the proliferation of BV-2 cells is reduced in presence of a low concentration of serum. (**J**) Whereas, in the presence of a low concentration of serum (1% FCS), the BV-2 cells adopted an elongated morphology, (**K**) they exhibited a rounded shape in presence of a high concentration of serum (10% FCS). Mean values ± SEM are shown, from *n* = 3–4 independent experiments, * *p* < 0.05, ** *p* < 0.01.

Serum is not found within the parenchyma of a healthy CNS. In pathological conditions, the rupture of the BBB allows the passage of large quantities of blood components. The exposure of microglia to serum leads to functional and transcriptomic modifications [11]. Therefore, serum represents an activation factor that we use in our experiments to mimic BBB disruptions. Here, we report the effect of decreasing concentrations of fetal calf serum (FCS) on cell survival, proliferation, morphology, and level of activation of the murine microglial cell line, BV-2. The exposure of BV-2 cells to reduced FCS concentrations is associated withan important loss of the cell viability determined by an MTT assay (Figure 1A) and corroborated by a 7-AAD staining, a dead cell exclusion dye (Figure 1B). Indeed, in comparison to BV-2 cells that are grown with 10% FCS, the MTT signal is decreased by 62.2% when the cells are with 2% FCS, by 74.3% with 1% FCS, and by 90.2% when the cells are grown in the absence of FCS. In a similar way, we quantified 90.9% of living cells (i.e., 7-AAD negative) in presence of 10% FCS, 58.1% with 2% FCS, 49.4% with 1% and only 27% in the absence of FCS. Thereafter, we focused our experiments on two FCS concentrations: 10% FCS, a high concentration that is associated to a great cell survival and supposed to induce an activation of BV-2, and 1% FCS, a low concentration reducing the survival to an acceptable level and supposed to drive BV-2 cells to a ‘non reactive’ state. To test this hypothesis, we applied the supernatant of BV-2 cells that were grown with 10% or 1% FCS on the oligodendroglial 158N cell line and analyzed their viability. Whereas, the supernatant of BV-2 grown with 1% FCS did not induce any loss of 158N cells (Figure 1C,D), the supernatant of BV-2 cultured in the presence of 10% FCS induced a decreased viability of 158N (Figure 1E,F). This toxicity indicates that 10% FCS is able to induce a harmful phenotype in BV-2 cells. To better know these two populations of BV-2, we further characterized them. Thus, the BV-2 cells grown with 1% FCS make one mitosis per 41 h, as assessed by the dilution of the dye CFSE (Figure 1G,I). In the presence of 10% FCS, the proliferative rate is significantly sped up to one division per 27 h (Figure 1H,I). The cellular morphology is commonly used as an indicator of the activation level that was adopted by microglia. While in the 1% FCS condition, the cells become adherent and develop processes (Figure 1J), in the 10% FCS condition, most of the cells are round-shaped (Figure 1K).

### 3.2. The BV-2 Cell Line Expresses Transcripts of GABA-A Receptors

**Figure 2:**
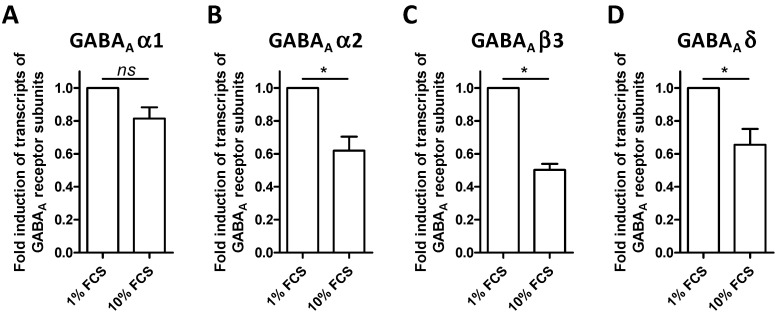
Gene expression of γ-aminobutyric acid-A (GABA-A) receptor subunits in BV-2 cells. Expression level of GABA-A receptor (**A**) α1, (**B**) α2, (**C**) β3, and (**D**) δ subunits in BV-2 cell line are determined by quantitative PCR. The experimental results represent the fold induction of mRNA expressed by BV-2 grown in presence of 10% FCS in comparison to BV-2 grown with 1% FCS after normalization to Ppia expression. Mean values ± SEM are shown, from *n* = 4 independent experiments, * *p* < 0.05.

ALLO seems to play a crucial role during cerebral diseases, notably thanks to its immunomodulatory activity. Most of the known effects exerted by this neurosteroid are mediated through the GABA-A receptors. To detect possible transcriptional regulations due to the serum concentrations used to cultivate BV-2 cells, the level of expression of specific GABA-A receptor mRNAs was investigated by quantitative PCR. We analyzed the expression of the following subunits normalized to Ppia expression: α1, α2, α4, β3, and δ. In our samples, the subunit α4 was not detected, whereas the other subunits were all found. The expression of the subunit α1 was unchanged between BV-2 cells grown with 1% FCS or 10% FCS (Figure 2A). On the contrary, there was a significant reduction in the expression of transcripts for the subunit α2, β3, and δ (Figure 2B–D). Similar results were obtained using other reference genes Hmbs and Pgk1 (data not shown).

### 3.3. Effect of ALLO on the Viability of BV-2 Cells

**Figure 3:**
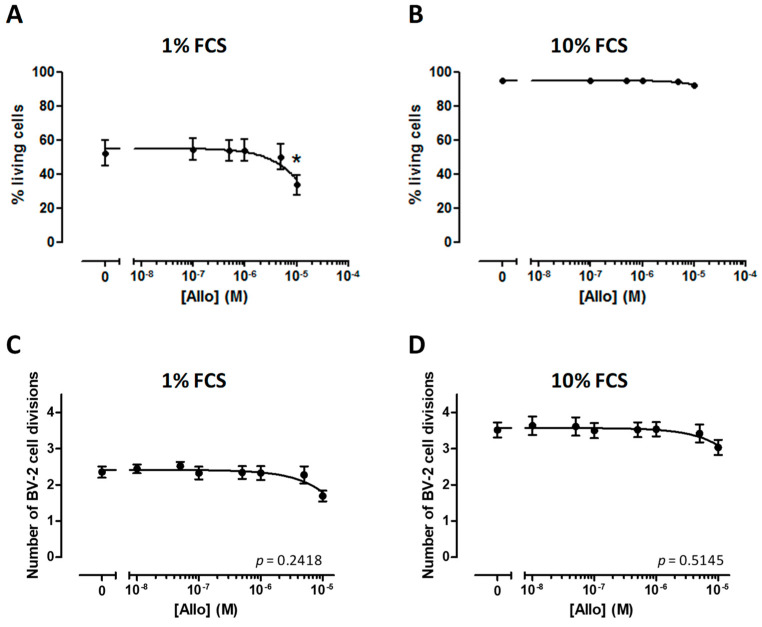
Effect of allopregnanolone (ALLO) on the viability and proliferation of BV-2 cells. (**A**,**B**) The viability of BV-2 cells was investigated by flow cytometry following incubation with graded concentrations of ALLO. (**C**,**D**) The dilution of CFSE showed that proliferation of BV-2 cells grown with 1% or 10% FCS is not modified in presence of ALLO. Mean values ± SEM are shown, from *n* = 4 independent experiments, * *p* < 0.05.

BV-2 cells that were grown in presence of 1% FCS exhibited a significant reduction of their viability only after incubation with 10 µM ALLO (Figure 3A). In contrast, none of the tested doses affected the viability of BV-2 cells cultured in the presence of 10% FCS (Figure 3B).

### 3.4. ALLO Does Not Modulate Proliferation of the BV-2 Cell Line

Neurosteroid ALLO has been reported to induce the proliferation of different cell types [37,38]. This parameter is important in the context of autoimmune diseases, where an uncontrolled proliferation of immune cells could exacerbate the inflammation. Thus, we analyzed the CFSE dilution in BV-2 cells by flow cytometry after four days of treatment with graded concentrations of ALLO. Whatever the dose of neurosteroid applied to BV-2 cells cultured in the presence of 1% FCS, the rate of cell division was unchanged (Figure 3C). A similar observation was done if BV-2 cells were grown with 10% FCS (Figure 3D).

### 3.5. ALLO Favors the Elongation of Microglial Processes

**Figure 4:**
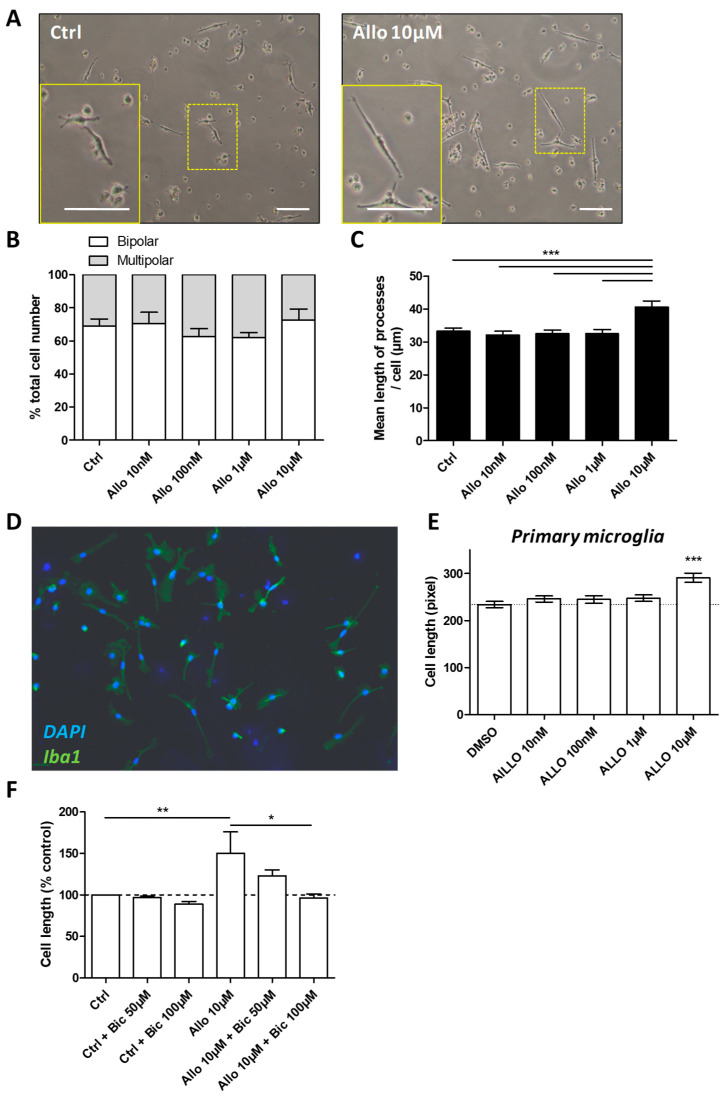
ALLO increases the length of microglial cells. (**A**) BV-2 cells grown in 1% FCS exhibited processes in both control and ALLO conditions. Scale bar = 50 µM. (**B**) The proportion of bipolar vs. multipolar BV-2 cells (i.e., cells with more than two processes) was not modified after a treatment with ALLO. Mean values ± SEM are shown, with *n* = 188, control cells; *n* = 141, 10 nM ALLO-treated cells; *n* = 164, 100 nM ALLO-treated cells; *n* = 172, 1 µM ALLO-treated cells; and *n* = 154, 10 µM ALLO-treated cells; from *n* = four independent experiments. (**C**) The mean length of the primary processes carried by BV-2 was increased after a treatment with ALLO (10 µM). Mean values ± SEM are shown, with *n* = 136, control cells; *n* = 105, 10 nM ALLO-treated cells; *n* = 97, 100 nM ALLO-treated cells; *n* = 106, 1 µM ALLO-treated cells; and *n* = 113, 10 µM ALLO-treated cells; from *n* = 4 independent experiments, *** *p* < 0.001. (**D**) Primary microglial cells that were grown in 1% FCS exhibited processes that had an increased length after incubation with ALLO (10 μM). Scale bar = 50 μM. (**E**) The mean length of primary microglial cells was increased after a treatment with ALLO (10 µM). Mean values ± SEM are shown, with *n* = 291, control cells; *n* = 391, 10 nM ALLO-treated cells; *n* = 289, 100 nM ALLO-treated cells; *n* = 306, 1 µM ALLO-treated cells; and, *n* = 274, 10 µM ALLO-treated cells; from five independent experiments, *** *p* < 0.001. (**F**) The effect of ALLO on BV-2 cell morphology is mediated by the GABA-A receptor. The application of bicuculline (Bic) before the treatment with ALLO (10 µM) abolished the ALLO-induced elongation of the processes carried by bipolar BV-2 cells. Mean values ± SEM are shown, from *n* = four independent experiments, * *p* < 0.05, ** *p* < 0.01.

To date, the effects of ALLO on microglia have not been deeply investigated. When grown in the presence of 1% FCS, BV-2 cells become adherent and develop processes (Figure 4A). In control conditions, among the BV-2 cells bearing processes, 69% were bipolar and 31% were multipolar exhibiting at least three extensions (Figure 4B). One day after the addition of ALLO, none of the tested doses was able to affect the proportion of bipolar vs. multipolar cells (Figure 4B). However, after treatment with 10 µM ALLO, BV-2 cells seemed to exhibit longer extensions (Figure 4A). The measurement of the processes borne by each cell revealed that the application of 10 µM ALLO led to an increase of 22% of their mean length (Figure 4C). This extension is found predominantly in bipolar cells (Supplementary Figure S1).

To note, the effect of ALLO on BV-2 morphology does not seem to be dependent of FCS concentration and, therefore, of BV-2 activation level. When these cells are cultured with 10% FCS, they barely develop processes, so we could not use the length of their extensions as an appropriate readout. Instead, we quantified the number of cells that showed a clear adherence to the plastic dish. Thus, in the presence of 10 µM ALLO, we could observe more cells exhibiting processes (Supplementary Figure S2A). Indeed, 14.2% of the cells were adherent in control conditions, whereas they were 19.2% after the addition of 10 µM ALLO (Supplementary Figure S2B).

Morphological analyses were also conducted on primary murine microglia cultured in 1% FCS. Immunostainings revealed that, in our experimental conditions, a high purity of microglia was achieved (Figure 4D). This was further confirmed by flow cytometry (Supplementary Figure S3). As expected, primary microglia exhibited processes when treated with vehicle. In the presence of 10 µM ALLO, the length of the cells was also significantly increased (Figure 4D,E). This result indicates that the BV-2 cell line and primary murine microglia both react in a similar manner to ALLO application. It also suggests that BV-2 cells may represent a valuable tool for dissecting the effect of ALLO on microglia.

ALLO is known to mediate effects via the GABA-A receptor. To investigate the implication of this receptor, bicuculline, a competitive antagonist of GABA-A receptors, was applied 30 min. before the incubation of the BV-2 cells with 10 µM ALLO. Bicuculline could prevent the elongation of the BV-2 cells in a dose-dependent manner (Figure 4F). The inhibition was complete with 100 µM bicuculline, a dosage that has already been used to modulate microglia [13,39]. This indicates that the observed cell length increase that was induced by ALLO was mediated by the GABA-A receptor.

### 3.6. ALLO Reduces the Motility of Microglia

**Figure 5:**
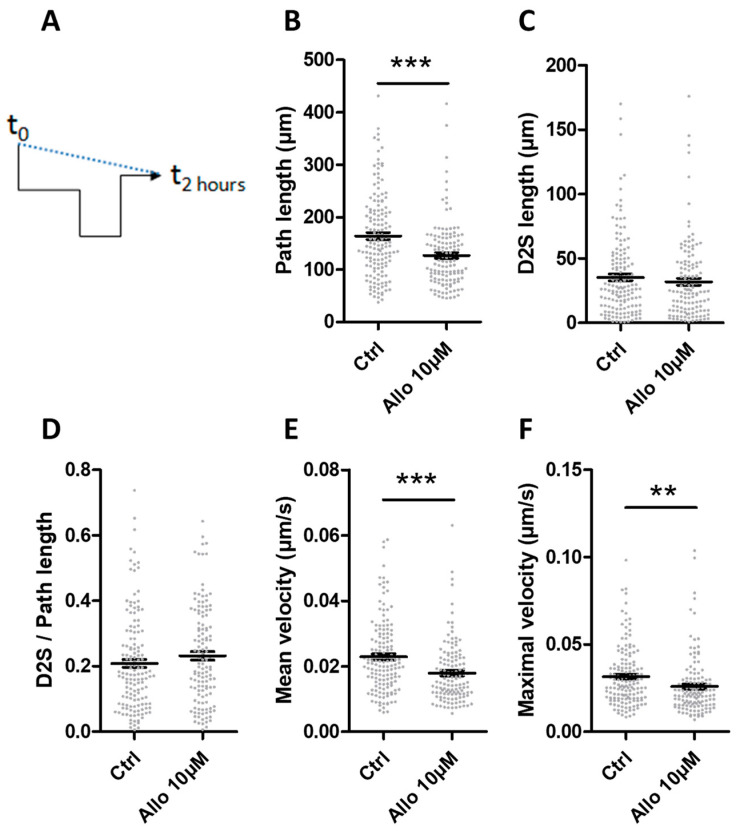
ALLO modifies the motility of BV-2 cells grown in a low concentration of serum. (**A**) The path length represents the total path traveled by the cells during the experiment (black line). D2S is the distance between the start and the end point (dotted blue line). (**B**) The path length and (**C**) the distance traveled between the first and the last position of the BV-2 cells (D2S length) were measured from time-lapse imaging. (**D**) The directionality (i.e., the ratio D2S length/path length), (**E**) the mean velocity and (**F**) the maximal velocity of BV-2 cells were subsequently determined. BV-2 cells were grown in 1% FCS. Mean values ± SEM are shown, with *n* = 146 control cells and *n* = 129 ALLO-treated cells, from four independent experiments, ** *p* < 0.01, *** *p* < 0.001.

Previous work that was realized by coauthors showed that a bipolar shape is associated with a more efficient migratory ability [40]. Because both primary microglia and BV-2 cells were more elongated following treatment with 10 µM ALLO, an effect on their motility was suspected. By using time-lapse imaging, the displacement of BV-2 cells grown in the presence of 1% FCS was analyzed in control conditions and after the addition of 10 µM ALLO. The movement of the nucleus was followed during 2 h. Unexpectedly, the cells that were treated with ALLO (that have the more elongated shape) appeared to be less motile. The mean path length underwent by BV-2 cells in the presence of ALLO was reduced by 22.6% during the acquisition time (Figure 5B). On the contrary, the distance traveled between the position at t0 and at t2hours was not modified by the treatment (Figure 5C). The analysis of the directionality, i.e., the ratio between the distance t0 to t2hours and the path length, indicated that the control BV-2 cells do not exhibit a persistent movement with a ratio of 0.21 (Figure 5D). This is consistent with the absence of any localized chemotactic signal in the culture conditions. The application of ALLO did not modify this parameter (Figure 5D). If we consider the mean and the maximum velocities (Vmax), the application of ALLO led to their significant reduction by 14.3% and 16.7%, respectively (Figure 5E,F).

### 3.7. Effect of ALLO on the Deleterious Activity Exerted by Reactive Microglial Supernatants on Oligodendrocytes

**Figure 6:**
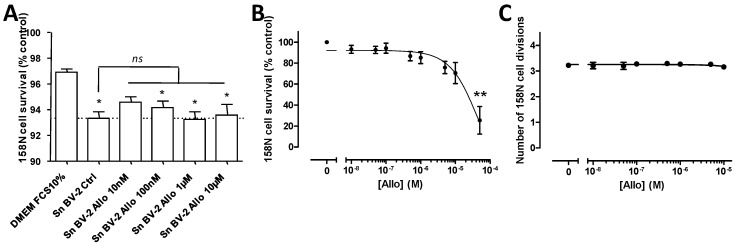
Effect of ALLO on reactive BV-2 cell derived supernatants in the modulation of oligodendrocyte survival. (**A**) The incubation of BV-2 with graded concentrations of ALLO from 10 nM to 10 µM did not modify the toxicity of their supernatants towards the oligodendrocyte 158N cell line. Mean values ± SEM are shown, from *n* = 4 independent experiments, * *p* < 0.05 versus ‘DMEM FCS10%’, *ns*: non-significant. (**B**) The effect of graded concentrations of ALLO on the viability and (**C**) on the proliferation of the 158N cells. Mean values ± SEM are shown, from *n* = 3–4 independent experiments, * *p* < 0.05, ** *p* < 0.01.

The supernatant of the activated BV-2 cells was able to alter the viability of the oligodendroglial 158N cell line in culture, as shown before (Figure 1E,F). We investigated whether the addition of ALLO on BV-2 cells that were grown with 10% FCS was able to counteract this deleterious effect. In presence of DMEM completed with 10% FCS, the 158N cells exhibit a high viability of 96% as determined by the exclusion of 7-AAD (Figure 6A). The addition of the supernatant from BV-2 cells led a significant decreased viability of 158N cells. The pre-incubation of BV-2 cells with graded concentrations of ALLO from 10 nM to 10 µM did not significantly improve the viability of 158N (Figure 6A). In order to know whether this neurosteroid could affect directly the viability of 158N cells, these cells were incubated in the presence of graded concentrations of ALLO. We could not detect any significant decrease of the 158N cell survival with the doses from 10 nM until 10 µM ALLO, doses used to counteract the deleterious activity of supernatants from reactive BV-2 cells (Figure 6B). Only the higher dose of 50 µM reduced the viability of 158N cells (Figure 6B). Of note, incubation with ALLO did not modify the proliferation of 158N cells (Figure 6C).

### 3.8. ALLO Modulates the Phagocytic Activity of Reactive Microglia

**Figure 7:**
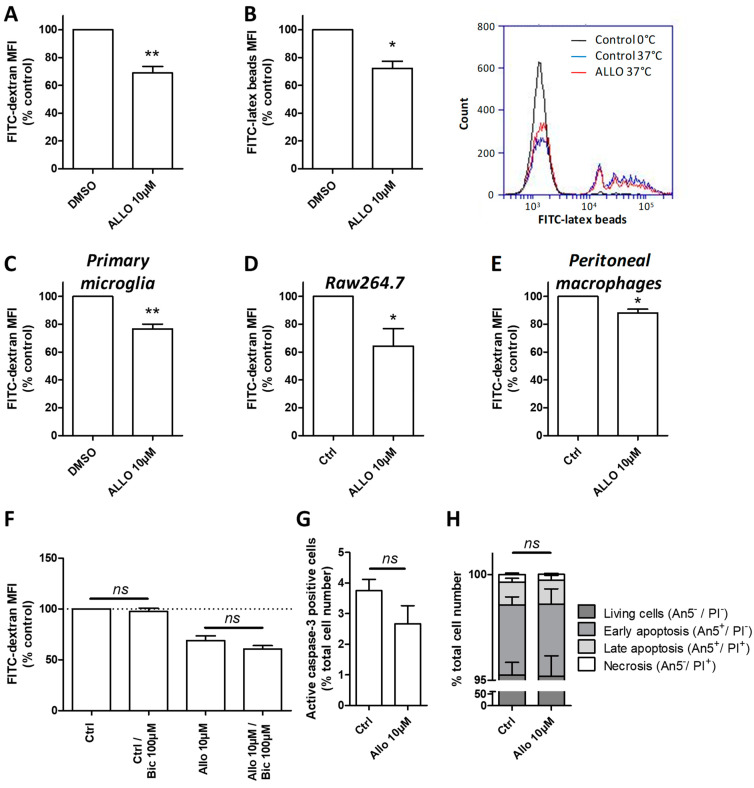
ALLO reduces the phagocytic activity of microglial cells grown in a high concentration of serum. (**A**) Application of 10 µM ALLO inhibits 70kDa FITC-dextran (**B**) as well as 1 µm FITC-latex beads uptake by BV-2 cells. (**C**) Primary murine microglia engulf also significantly less FITC-dextran after treatment with ALLO. (**D**) Murine macrophages such as the Raw 264.7 cell line or (**E**) peritoneal macrophages are also sensitive to ALLO that reduces significantly their FITC-dextran uptake. Mean values ± SEM are shown, from *n* = 4–6 independent experiments, * *p* < 0.05. (**F**) Effect of bicuculline on the reduced phagocytic activity induced by 10 µM ALLO on BV-2 cells. (**G**) The treatment of BV-2 with 10 µM ALLO does not induce apoptosis, as revealed by caspase-3 intracellular staining or (**H**) annexin5 (An5)/propidium iodide (PI). Mean values ± SEM are shown, from *n* = 3–4 independent experiments, * *p* < 0.05, ** *p* < 0.01.

ALLO, besides its well-known neuroprotective action, can also modulate immune cell activity [41]. Phagocytosis represents an immunological function exerted by microglia that is highly dependent on the dynamics of the cytoskeleton. Moreover, serum exposure is known to enhance phagocytosis [11]. To investigate the potential action of ALLO on phagocytosis, microglial cells were incubated either with 70kDa FITC-dextran or 1 µm FITC-latex beads. The internalization of FITC-dextran was significantly reduced when BV-2 cells were pre-treated with 10 µM ALLO (Figure 7A). The uptake of larger particles, like latex beads, was also impaired by ALLO treatment, as demonstrated by the decreased MFI that was measured by flow cytometry (Figure 7B). The proportion of cells having engulfed latex beads is also decreased after the application of ALLO.

Very interestingly, a similar finding was observed with primary microglia treated with 10 µM ALLO. Indeed, these cells exhibited a significantly lower capacity to take FITC-dextran (Figure 7C). Microglia are considered to be the resident macrophage population of the CNS. FITC-dextran uptake was also analyzed in the Raw264.7 cell line and in peritoneal macrophages in order to assess whether the inhibitory action of ALLO on phagocytic activity was general on other macrophage populations. The addition of 10 µM ALLO on Raw264.7 cells led to a significantly decreased internalization of FITC-dextran in comparison to control cells (Figure 7D). More interestingly, macrophages directly derived from the peritoneal cavity of adult mice also exhibited a reduced phagocytic capacity after the application of ALLO (Figure 7E). These reproducible results obtained with various cells indicate the robust effect of ALLO on phagocytosis.

Bicuculline was used to understand the mechanism leading to the decreased uptake by microglia. A pre-treatment with this GABA-A antagonist was not able to prevent the inhibited FITC-dextran uptake produced by 10 µM ALLO (Figure 7F). This result suggests that this effect was independent of GABA-A signaling. Moreover, to exclude that this inhibitory effect generated by 10 µM ALLO was due to a potential toxic effect, we analyzed apoptotic and necrotic markers. The incubation of BV-2 cells with 10 µM ALLO did not modify the percentage of caspase-3 positive cells, as measured by flow cytometry (Figure 7G). Using the markers PI and annexin-5, we could also determine the ratio of cells in healthy condition, early or late apoptosis, and undergoing necrosis (Figure 7H). In the control culture, the vast majority of BV-2 cells consisted of living cells negative for both PI and annexin-5. Less that 5% accounted for dead and dying cells (Figure 7H). The application of 10 µM ALLO did not alter the ratios of the different populations (Figure 7H). Thus, ALLO modified the phagocytic capacity of BV-2 cells independentof any deleterious effect.

### 3.9. ALLO Inhibits the Inappropriate and Unexpected Phagocytosis of Oligodendrocytes by Reactive Microglia

**Figure 8:**
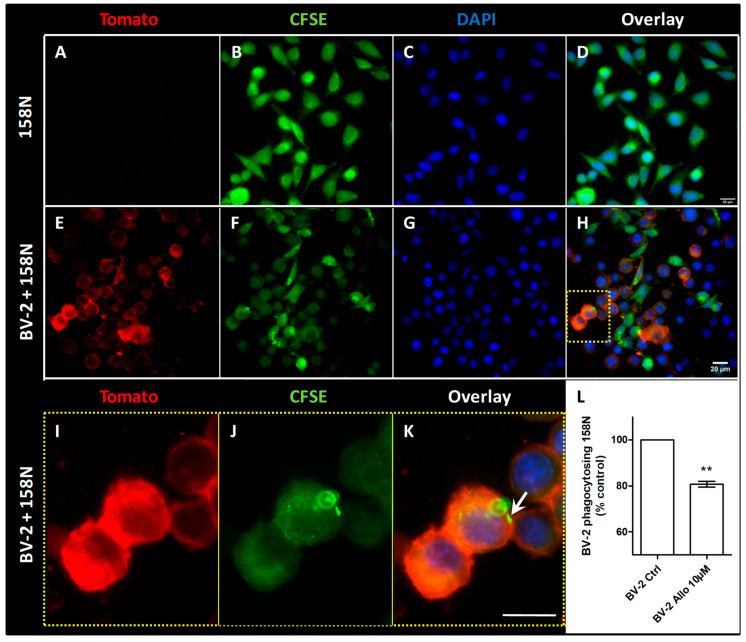
ALLO inhibits the inappropriate phagocytosis of oligodendroglial 158N cells by microglial BV-2 cells. (**A**–**K**) Microscopic observation of 158N cells either alone (**A**–**D**) or in coculture with BV-2 cells (**E**–**K**). BV-2 cells express the red fluorescent protein Tomato (**E**,**H**,**I**,**K**) and 158N cells are previously stained with the green fluorescent compound CFSE (**B**,**D**,**F**,**H**,**J**,**K**). The nuclei are observed by DAPI (**C**,**D**,**G**,**H**,**K**). The fluorescent signals detected in each channel are presented overlaid (**D**,**H**,**K**). The BV-2 cells observed in the yellow dashed box of the microphotograph (**H**) are shown with a higher magnification (**I**–**K**). One BV-2 cell contains green fluorescence derived from a phagocytized 158N cell (arrow). Scale bar = 20 µm. (**L**) Flow cytometric measurement of red fluorescent BV-2 cells having phagocytized green fluorescent 158N. Mean values ± SEM are shown, from *n* = five independent experiments, ** *p* < 0.01.

In order to investigate whether the activation of microglia could lead to an inappropriate phagocytosis of oligodendrocytes, we designed an in vitro model where activated BV-2 cells grown in presence of 10% FCS and expressing the red fluorescent molecule tomato were co-cultured with 158N cells that were previously stained with the green fluorescent CFSE (Figure 8). Oligodendroglial 158N are adherent cells presenting short elongations and are devoid of red fluorescence (Figure 8A–D). In the presence of BV-2 cells, they exhibit a similar morphology (Figure 8E–H). The activated BV-2 cells are mostly rounded (Figure 8E–K). Some BV-2 cells contain green fluorescence derived from phagocytized oligodendroglial 158N (Figure 8I–K). When the BV-2 cells were previously grown in the presence of 10% FCS, 2.07% of these cells were found to have phagocytized 158N cells. The pre-treatment of BV-2 cells with 10 µM ALLO significantlyreduced the inappropriate phagocytosis of 158N cells (Figure 8L).

## 4. Discussion

We used both murine primary microglia and the BV-2 cell line to analyze the effects of neurosteroid ALLO on microglia in physiological and pathological conditions. BV-2 cells represent an effective substitute for microglial primary cultures [42]. Indeed, when exposed to lipopolysaccharides, 90% of the genes induced by primary microglia were also induced by BV-2 cells. Moreover, BV-2 cells are commonly employed as an in vitro model of neuroinflammation. Thus, we used BV-2 cells to screen multiple parameters in order to investigate the effects of ALLO on microglial cells. Furthermore, to increase the pathophysiological significance of our findings, the relevant results that were obtained with BV-2 cells were validated in primary microglial cultures that are conventionally well accepted as the most reliable cell model to reproduce in vitro the conditions occurring in vivo.

Serum is not found within the parenchyma of a healthy CNS. In pathological conditions, the rupture of the BBB allows for the passage of large quantities of blood components. The exposure of microglia to serum leads to functional and transcriptomic modifications in both BV-2 cells and primary microglia [11,43]. For instance, when cultured with a reduced concentration of FCS, both BV-2 cells and primary microglia adopt a process-bearing morphology that is typical of highly ramified surveillant microglia. In contrast, in the presence of a high concentration of serum, BV-2 and primary microglia exhibit a round amoeboid shape, a representative morphology of fully activated microglia typically found in the injured brain [11,43]. Phagocytic activity that is exerted by microglia is also related to FCS concentration. This first line of defense mechanism is highly active in primary microglia grown in the presence of 10% FCS [44]. In contrast, in the absence of serum, primary microglial cells barely engulf any particle [11]. The serum-induced microglial phagocytosis may be due to signals that are normally excluded from the CNS environment and, therefore, linked to BBB integrity [44]. Indeed, an in vivo model of neuroinflammation in which the BBB is maintained, like peripheral LPS injection, did not demonstrate enhanced phagocytosis [45]. In contrast, debris uptake by microglia has been documented in neuropathologies, where BBB is compromised, such as stroke or multiple sclerosis [46,47]. Phagocytosis is now recognized as a critical mechanism for neuroprotection and regeneration that is regulated by serum factors. Moreover, we could demonstrate that the supernatant derived from BV-2 cells cultured with high serum was harmful to the 158N oligodendrocyte cell line, suggesting that they may have secreted deleterious factors. In contrast, no modification of 158N oligodendrocyte viability was observed when they were incubated with the supernatant from BV-2 cells grown in the presence of a low concentration of FCS. Therefore, serum represents a valuable activation factor to mimic compromised BBB found in neuropathologies, such as multiple sclerosis. Thus, we considered having two experimental conditions: BV-2 cells in ‘resting’ state to mimic the physiological condition (i.e., grown with 1% FCS), and reactive BV-2 cells to mimic a pathological state (i.e., grown with 10% FCS).

Neurosteroid ALLO is known to exert neuroprotection in numerous neurological diseases, in particular MS [20]. To date, the underlying mechanisms are poorly understood, especially the ones leading to the modulation of microglial activity. In a physiological microenvironment, the microglial cells have a small soma bearing long and thin extensions. Upon activation, a gradual morphological transformation is marked by the retraction of cellular processes. For this reason, the analysis of microglial morphology is widely used to quantify their activation when their involvement is investigated in neurological diseases, such as stroke [48,49] or multiple sclerosis [50,51]. In this report, we found that the addition of ALLO led to morphological modifications of BV-2 cells and primary microglia, which exhibited longer processes. This morphological modification may represent the shift of the cells towards a more resting state. Interestingly, we also report that this elongated shape is associated with a reduced migratory capacity. The somata of resting microglial cells remain fixed, as revealed by in vivo two-photon microscopy [4]. In contrast, the microglial cells have been shown to exhibit a migratory activity in neuropathological conditions. For instance, they migrate in the direction of a forebrain stab lesion [52] or towards newly forming plaques in an animal model of Alzheimer’s disease [53]. Interestingly, a similar morphological modification has been evidenced while using Schwann cells. In the presence of 1 µM ALLO, these cells adopt the typical spindle-shaped morphology [54]. The actin rearrangements that are induced by ALLO involved the signaling pathway Src/FAK (Src = proto-oncogene tyrosine-protein kinase; FAK: focal adhesion kinase) and GABA-A dependent mechanisms [54]. The elongation of extensions borne by BV-2 reported appeared here to be mainly mediated by the GABA-A receptor, as it was completely reversed by the application of bicuculline, a competitive antagonist at the GABA-binding site of the GABA-A receptor.

Phagocytosis constitutes a fundamental activity of innate immune cells. In multiple sclerosis, myelin phagocytosis represents a pathological hallmark. By using two different substrates in the present work, we demonstrate an inhibitory activity being exerted by ALLO on microglial phagocytic function. More interestingly, in a coculture experiment, we show that phagocytosis of oligodendrocytes, the cells forming the myelin sheath in the CNS, is also decreased after the treatment of microglia by ALLO. Impaired expressions of homeostatic molecules have been reported to point to the onset of uncontrolled phagocytosis by microglia in MS lesions [55]. Thus, the modulation of phagocytosis appears critical to improve this pathology.

GABA-A receptors are pentameric ligand-gated ion channels that can be composed with various subunits (α, β, γ, δ, ε, θ, π, and ρ) and they exhibit a variety of allosteric binding sites [56]. Some pharmacological properties are critically linked to the subunit combination that is found within the receptor. For instance, the exclusive extrasynaptic presence of GABA-A receptors formed with α-β or α-β-δ subunits is known to exert a tonic inhibition of neurons [57]. Murine microglial cells were shown to express the mRNA of 15 subunits (α1–5, β1–3, γ1–3, δ, ε, ρ1–2) [18] and exhibit functionally active GABA-A receptors [13]. Regarding the GABA-A receptor subunits that are expressed by BV-2 cell line, we found the expression of mRNA coding for α1, as previously described [39], as well as α2, β3, and δ. On the contrary to α1, the transcripts of α2, β3, and δ were more expressed in cells that were grown with 1% FCS. The subunit α2 has been recently found to be implicated in the inflammatory activation of monocyte/macrophage RAW264.7 cell line following LPS stimulation [58]. In particular, we investigated the expression of the δ subunit because neurosteroids have preferential affinity for δ-containing GABA-A receptors [59]. Moreover, δ subunit-containing receptors exhibit a high affinity for GABA and can be activated by low nanomolar concentrations of GABA that spill over the synaptic cleft or are released by glial cells [60]. Interestingly, the subunit β3, reported to form homo-oligomeric GABA-A receptors, also contains a steroid-binding site at the transmembrane domain 3 [61]. Thus, a higher expression of β3 and δ GABA-A subunits in BV-2 cells that were grown in 1% FCS might lead to a higher affinity of ALLO for the GABA-A receptors. In the present report, we could demonstrate that the same dose of 10 µM ALLO was able to modify the morphology of BV-2 cells grown with 1% FCS as well as the phagocytic function of BV-2 cells grown with 10% FCS. Accordingly, there is no mismatch of the efficient dose that is able to modify either morphology or phagocytosis despite the potential modified expression of GABA-A receptor subunits in these cells. We did not evaluate the transcription of the other subunits that might be upregulated as a compensatory mechanism. Additionally, we did not assess whether these transcriptomic modulations would be translated at the protein level. Thus, we cannot conclude on the total concentration of GABA-A receptors that would be found at the microglial membrane surface, either upon 1% or 10% FCS.

The activity of ALLO is known to be notably mediated through the GABA-A receptor by a concentration-dependent mechanism [62]. The only significant effects of ALLO that are shown in the present study are observed with the concentration 10 µM. Interestingly, a concentration of 10 µM ALLO has previously been reported to decrease NO secretion by BV-2 cells [41] or reduce pro-inflammatory cytokine transcription by primary rat microglial cells [63]. At low nanomolar concentrations, ALLO acts as a positive allosteric modulator enhancing the action of the natural ligand GABA, whereas, at micromolar concentrations, this neurosteroid directly activates the receptors even in the absence of GABA [60,64,65]. This profile is explained by the presence of two distinct neurosteroid binding sites in the GABA-A receptor’s transmembrane domains, which are distinct from the GABA binding site. Neurosteroids at nanomolar concentrations potentiate GABA responses from the ‘potentiation site’ that is formed by the α-subunit transmembrane domains. Micromolar concentrations of neurosteroids directly activate GABA-A receptor by binding to the ‘activation’ site located between α and β subunits. Thus, significant GABA-A receptor activation by neurosteroids relies on occupancy of both the activation and potentiation sites [60]. Moreover, using exogenous systems like HEK2293 cells, neurosteroids were shown to enhance the current that is evoked by low concentrations of GABA, but not the current evoked by saturating concentrations of GABA [66]. Thus, neurosteroids increase the GABA efficacy of these receptors by converting GABA from a partial to full agonist [67]. It would be then interesting to assess and control GABA concentrations in our conditions of culture to investigate whether it is responsible of the different responses that were observed after bicuculline treatment.

Whereas the morphological extension induced by ALLO was completely abolished by pre-treatment of the cells with bicuculline, this GABA-A antagonist did not modify the decreased phagocytic activity that was observed after the addition of ALLO. Bicuculline is considered as a competitive antagonist of GABA-A receptors, which means that it competes to occupy the GABA binding site. Bicuculline does not compete for binding at the neurosteroid sites of GABA-A receptors [68]. Unlike GABA, bicuculline cannot induce the rotation of β subunits and, therefore, stabilizes the closed channel pore [69], with a complete blocking effect at the concentration 100 µM [68]. Of prime interest, the inhibitory action of thiopental on monocyte phagocytosis was shown to only be mildly prevented by GABA-A antagonists: bicuculline and picrotoxin [70]. At the opposite, in this same study, bicuculline completely abolished the inhibitory effect of thiopental on monocyte migration [70]. These observations suggest that drugs, like anaesthetic compounds or neurosteroids, which can bind to GABA-A receptors, could also act via other mechanisms or be dependent of other factors.

Besides the mediation through GABA-A receptors, the activity of ALLO can also rely on other targets such as L-type voltage-gated calcium channels (VGCC), liver X receptors (LXRs), pregnane xenobiotic receptors (PXRs), or ATP-gated P2X receptor channels. Transcripts for l-type VGCC isoforms are detectable in immune cells [71], as well as in murine microglia [18] and BV-2 cells [72]. LXRs are members of the nuclear hormone receptor superfamily existing in two forms, the inducible LXR α and the ubiquitous LXR β. Both of the isoforms are expressed by CD4^+^ T cells and macrophages, where their activation reduces proinflammatory cytokine expression [73,74]. In BV-2 cells, the expression of LXR α has been associated to the anti-inflammatory activity of taraxasterol [75]. PXR is expressed in monocyte/macrophage cells, where they participate in an anti-inflammatory pathway [76]. P2X receptors expression is restricted to the P2X 4 and P2 X7 receptor subtypes in microglia [77,78]. We can discard the reconversion of ALLO toward 5α-dihydroprogesterone, as murine microglia seem not to express 3α-hydroxysteroid oxydo-reductase, the enzyme that allows this reaction [79].

Steroid hormones are known to modulate cell proliferation. ALLO has also been reported to increase the proliferation of different cell types, such as postnatal hippocampal cells [37], SH-SY5Y cells [38], or Schwann cells [80]. Neither the microglial BV-2 nor the oligodendroglial 158N cell line used in this study showed a modified proliferation after the administration of ALLO in the range of 10 nM to 10 µM (Figure 3C,D or Figure 6C). Accordingly, ALLO also failed to promote the proliferation of oligodendrocyte precursors in cultured cerebellar slices of seven-day-old rats [81].

The present data describe the effects of ALLO on microglial morphology and phagocytic activity. These results constitute direct evidence representing a strong and interesting starting point for further analysis to determine the underlying molecular mechanisms that lead to the observed modulatory effects on microglia in order to strengthen “neurosteroid-based approach” for the development of effective strategies against neurological disorders. Moreover, the investigations of microglial synthesis and secretion of inflammatory mediators, such as cytokines and reactive oxygen species, upon ALLO treatment would represent attractive prospects.

## 5. Conclusions

**Figure 9:**
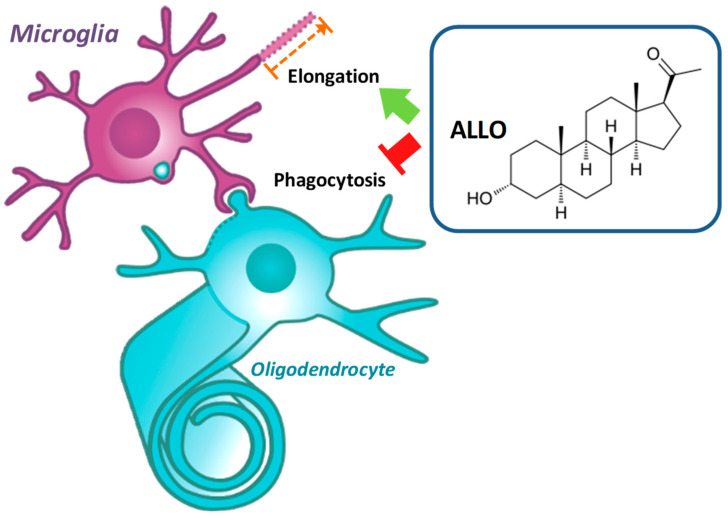
Graphical abstract demonstrates that the natural neurosteroid allopregnanolone (ALLO) inhibits oligodendrocyte phagocytosis by microglia. ALLO modifies also microglial morphology by increasing the length of cell processes.

To conclude, as displayed in Figure 9, we have shown that microglia express transcripts of GABA-A receptor and that they are sensitive to ALLO. In physiological condition, ALLO could represent an important molecule maintaining microglia in an inactivated state that is characterized by a ramified morphology with long and thin processes. In the pathological condition, microglia are getting activated to remove debris. An inappropriate and exacerbated activation of microglia may lead to an uncontrolled phagocytic activity of viable cells. Therefore, the restoration of normal ALLO concentrations in the CNS of MS patients deficient for this neurosteroid may prevent collateral victims and participate in the downregulation of the auto-reactive immune response that is directed against myelin.

## Figures and Tables

**Table 1:** Sequences of the oligonucleotides used for real-time PCR amplification.

mRNA Target (Gene Name)	Forward Primer (5’-3’)	Reverse Primer (3’-5’)	Amplicon (bp)
GABA-A R subunits			
α1 (GabrA1)	AAAAGCGTGGTTCCAGAAAA	GCTGGTTGCTGTAGGAGCAT	84
α2 (GabrA2)	GCTACGCTTACACAACCTCAGA	GACTGGCCCAGCAAATCATACT	115
β3 (GabrB3)	GGGACCCCCGAAGTCGGGTCT	GAGCGTAAACGACCCCGGGAA	100
δ (GabrD)	TCAAATCGGCTGGCCAGTTCCC	GCACGGCTGCCTGGCTAATCC	145
Reference genes			
Glyceraldehyde-3-phosphate dehydrogenase (Gapdh)	AGGTCGGTGTGAACGGATTTG	TGTAGACCATGTAGTTGAGGTCA	123
Peptidylprolyl isomerase A (Ppia)	AGGGTTCCTCCTTTCACAGAATT	TGCCATTATGGCGTGTAAAGTC	72
Phosphoglycerate kinase 1 (Pgk1)	ATTCTGCTTGGACAATGGAGC	AGGCATGGGAACACCATCA	76

Bp, base pair.

## Data Availability

The data that support the findings of this study are available from the corresponding author upon reasonable request.

## Supplementary Materials

The following are available online at https://www.mdpi.com/2073-4409/10/3/698/s1, Figure S1: ALLO increases the length of BV-2 cells grown in a low concentration of serum. (A) The lengths of the processes borne by bipolar and (B) multipolar BV-2 cells were split apart., Figure S2: ALLO increases the adherence of BV-2 cells grown in a high concentration of serum. (A) BV-2 cells grown in 10% FCS exhibited a rounded shape and seemed more adherent after incubation with ALLO (10 µM). Scale bar = 10 µM. (B) The incubation of BV-2 cells grown in 10% FCS with ALLO increased the percentage of adherent cells (i.e., cells exhibiting processes). Mean values ± SEM are shown, from *n* = 3 independent experiments, * *p* < 0.05., Figure S3: Purity of primary microglia culture. Flow cytometry analysis of primary microglia after CD11b and CD45 stainings.
